# The impact of cancer on subsequent chance of pregnancy: a population-based analysis

**DOI:** 10.1093/humrep/dey216

**Published:** 2018-06-15

**Authors:** Richard A Anderson, David H Brewster, Rachael Wood, Sian Nowell, Colin Fischbacher, Tom W Kelsey, W Hamish B Wallace

**Affiliations:** 1 MRC Centre for Reproductive Health, Queen’s Medical Research Institute, University of Edinburgh, 47 Little france Crescent, Edinburgh, UK; 2 Scottish Cancer Registry, Information Services Division, NHS National Services Scotland, 1 South Gyle Crescent, Edinburgh, UK; 3 Information Services Division, NHS National Services Scotland, 1 South Gyle Crescent, Edinburgh, UK; 4 eData Research & Innovation Service (eDRIS), Information Services Division, NHS National Services Scotland, Edinburgh, 1 South Gyle Crescent, Edinburgh, UK; 5 Farr Institute Scotland, Nine Edinburgh Bioquarter, Little France Road, Edinburgh, UK; 6 School of Computer Science, University of St. Andrews, North Haugh, St. Andrews, UK; 7 Department of Oncology and Haematology, Royal Hospital for Sick Children, Sciennes Road, Edinburgh, UK

**Keywords:** epidemiology, fertility preservation, pregnancy, cancer, cancer survivor, pregnancy outcome

## Abstract

**STUDY QUESTION:**

What is the impact of cancer in females aged ≤39 years on subsequent chance of pregnancy?

**SUMMARY ANSWER:**

Cancer survivors achieved fewer pregnancies across all cancer types, and the chance of achieving a first pregnancy was also lower.

**WHAT IS KNOWN ALREADY:**

The diagnosis and treatment of cancer in young females may be associated with reduced fertility but the true pregnancy deficit in a population is unknown.

**STUDY DESIGN, SIZE, DURATION:**

We performed a retrospective cohort study relating first incident cancer diagnosed between 1981 and 2012 to subsequent pregnancy in all female patients in Scotland aged 39 years or less at cancer diagnosis (*n* = 23 201). Pregnancies were included up to end of 2014. Females from the exposed group not pregnant before cancer diagnosis (*n* = 10 271) were compared with general population controls matched for age, deprivation quintile and year of diagnosis.

**PARTICIPANTS/MATERIALS, SETTING, METHODS:**

Scottish Cancer Registry records were linked to hospital discharge records to calculate standardized incidence ratios (SIR) for pregnancy, standardized for age and year of diagnosis. Linkage to death records was also performed. We also selected women from the exposed group who had not been pregnant prior to their cancer diagnosis who were compared with a matched control group from the general population. Additional analyses were performed for breast cancer, Hodgkin lymphoma, leukaemia, cervical cancer and brain/CNS cancers.

**MAIN RESULTS AND THE ROLE OF CHANCE:**

Cancer survivors achieved fewer pregnancies: SIR 0.62 (95% CI: 0.60, 0.63). Reduced SIR was observed for all cancer types. The chance of achieving a first pregnancy was also lower, adjusted hazard ratio = 0.57 (95% CI: 0.53, 0.61) for women >5 years after diagnosis, with marked reductions in women with breast, cervical and brain/CNS tumours, and leukaemia. The effect was reduced with more recent treatment period overall and in cervical cancer, breast cancer and Hodgkin lymphoma, but was unchanged for leukaemia or brain/CNS cancers. The proportion of pregnancies that ended in termination was lower after a cancer diagnosis, and the proportion ending in live birth was higher (78.7 vs 75.6%, CI of difference: 1.1, 5.0).

**LIMITATIONS, REASONS FOR CAUTION:**

Details of treatments received were not available, so the impact of specific treatment regimens on fertility could not be assessed. Limited duration of follow-up was available for women diagnosed in the most recent time period.

**WIDER IMPLICATIONS OF THE FINDINGS:**

This analysis provides population-based quantification by cancer type of the effect of cancer and its treatment on subsequent pregnancy across the reproductive age range, and how this has changed in recent decades. The demonstration of a reduced chance of pregnancy across all cancer types and the changing impact in some but not other common cancers highlights the need for appropriate fertility counselling of all females of reproductive age at diagnosis.

**STUDY FUNDING/COMPETING INTEREST(S):**

This study was funded by NHS Lothian Cancer and Leukaemia Endowments Fund. Part of this work was undertaken in the MRC Centre for Reproductive Health which is funded by the MRC Centre grant MR/N022556/1. RAA has participated in Advisory Boards and/or received speaker’s fees from Beckman Coulter, IBSA, Merck and Roche Diagnostics. He has received research support from Roche Diagnostics, Ansh labs and Ferring. The other authors have no conflicts to declare.

## Introduction

Continuing advances in therapy mean that many girls and young women can now expect long-term survival following a diagnosis of cancer, and thus there exists a rapidly increasing population of survivors of childhood and young adulthood cancer (Skinner *et al.*, 2006). Increasing importance is therefore placed on the quality of their survivorship and their risk of ‘late effects’ from their successful treatment (Oeffinger *et al.*, 2006; Armstrong *et al.*, 2016). The possible impact on fertility is one of the consequences of cancer treatment of greatest importance to patients (Peate *et al.*, 2009).

The adverse effects of cancer treatment on fertility in both men and women have been recognized for many years, and the importance and establishment of fertility preservation as part of current medical practice is recognized in international guidelines (Loren *et al.*, 2013; National Institute for Clinical Excellence, 2013). What is less clear, however, is the overall extent of post-cancer loss of fertility within a population, as investigation of this issue has often focused on specific diagnoses or otherwise selected patient groups (Bramswig *et al.*, 2015; Armuand *et al.*, 2017). The US Childhood Cancer Survivors Study (CCSS), which is based on self-reported questionnaires, has provided detailed analyses of fertility and pregnancy outcomes in relation to diagnosis and treatment using siblings as controls (Signorello *et al.*, 2006; Green *et al.*, 2009; Chow *et al.*, 2016). However, this and the British Childhood Cancer Survivors Study (BCCSS) (Reulen *et al.*, 2009) are confined to those diagnosed before the age of 21 and 15 years respectively, with limited data on the extent of reduced fertility and pregnancy outcome in unselected populations (Chiarelli *et al.*, 1999; Clark *et al.*, 2007; Winther *et al.*, 2009a; Haggar *et al.*, 2014). Additionally, studies of reproductive function in adult women have often used amenorrhoea or premature ovarian insufficiency (POI) as the key outcome (Swerdlow *et al.*, 2014; Jacobson *et al.*, 2016) which may not closely reflect the experience of failure to conceive in these patients (Letourneau *et al.*, 2012; Barton *et al.*, 2013), additionally impacted by social, psychological and sexual effects of cancer and its treatment (Ganz *et al.*, 1998; Howard-Anderson *et al.*, 2012).

In Scotland, the availability of linkable databases of cancer registrations and pregnancy-related outcome records offers the opportunity to study whether women achieve pregnancy after a cancer diagnosis on a population basis.

## Materials and Methods

### Study population

Female patients with a record of a first incident cancer diagnosed below the age of 40 years between 1981 and 2012 in Scotland were identified from the Scottish Cancer Registry and linked to national general and maternity hospital discharge records to ascertain subsequent pregnancies (miscarriage, termination of pregnancy, or delivery of a still or live born infant) up until the end of 2014. Linkage to subsequent death records up to the end of 2014 was also performed (see Supplementary Information Tables S1–S3 for ICD codes). Record linkage involved deterministic matching based on the Community Health Index (CHI) number, a unique identifying number used on all patient records in Scotland. Individuals were assigned to population-weighted fifths of deprivation scores (Morris and Carstairs, 1991) by applying 1991 and 2001 census-derived scores to the periods of diagnosis 1981–1995 and 1996–2012, respectively. This is based on small area of residence, and is derived from four variables collected at each decennial census: social class, unemployment, overcrowding and car ownership.

Patients treated with radiotherapy and with chemotherapy in the first 2 years following the cancer incidence date from 1997 onwards were identified from Scottish Cancer Registry records; prior to 1997, acute hospital discharge records were used to identify any radiotherapy or chemotherapy treatments (see Supplementary Information Tables S1–S3). As these records do not contain all episodes of radiotherapy or chemotherapy treatment, any patients with no matching treatment records were recorded as Not Known.

### Ethical approval

The study was approved by the Privacy Advisory Committee of the National Health Service National Services Scotland—study reference number XRB13215.

### Standardized incidence ratio of subsequent pregnancy for all women with cancer

We compared the total number of pregnancies in the exposed group after cancer diagnosis to the number expected based on pregnancy rates in the general population from the date of cancer incidence to the date of death or 31 December 2014, whichever occurred first. Indirectly standardized incidence ratios (SIR) of pregnancy were calculated, standardized for age, deprivation quintile and calendar year of diagnosis. Pregnancy rates for the general population were calculated using mid-year population estimate denominator data sourced from National Records of Scotland. The overall impact of each cancer diagnostic group was calculated from the number of women with each diagnosis and its impact, as a proportion of the total pregnancy deficit.

### Subsequent first pregnancy for women nulliparous at cancer diagnosis

The second part of the study selected only women from the exposed group who had not been pregnant before their cancer diagnosis (or within 6 months of the date of cancer diagnosis to ensure exclusion of women diagnosed during pregnancy). Data on previous pregnancies were available from 1981 onwards. An unexposed control group, similarly required to have had no pregnancy outcome events before or within 6 months of the matching date, was created from the general population using the CHI database which includes all patients registered with a General Practitioner in Scotland. Controls were matched on age, deprivation quintile and year of cancer diagnosis. For exposed patients diagnosed between 1981 and 1997, the deprivation category of controls as at 1997 was used for matching because address details before 1997 (required to assign deprivation status) were not known. Three controls were selected for every member of the exposed group. Of the 30 813 controls, two were subsequently removed from the analysis due to incorrect linkage.

The primary outcome was the first pregnancy event (miscarriage, termination or delivery) occurring at least 6 months after the date of cancer incidence, or the corresponding date in matched controls. We also examined the proportions of pregnancies ending in miscarriage, termination, still birth or live birth, as well as the still birth and infant death rates.

Cumulative incidence curves were produced for each cancer type showing the cumulative incidence of first pregnancy and, separately, death over follow up time for the exposed cases and the controls (Scrucca *et al.*, 2007). Because mortality was an important competing risk, Fine and Gray competing risk models (Fine and Gray, 1999) were used to calculate sub-distribution hazard ratios (HRs) and 95% CI for pregnancy. Four models were run to examine variations in the association between cancer and pregnancy by (i) duration of follow-up time (since hazards were not proportional over time); (ii) age group at diagnosis of cancer; (iii) deprivation quintile; and (iv) period of diagnosis of cancer. Both unadjusted HRs and HRs adjusted for age group at diagnosis, deprivation quintile, period of diagnosis and cancer type as appropriate were produced.

Three further models were run: (i) HRs for pregnancy for the different cancer types relative to the entire control group controlling for other factors. (ii) An extension of this model including an interaction term between period of diagnosis and cancer type to generate adjusted HRs by period of diagnosis for pre-specified cancer types of interest: leukaemia, Hodgkin lymphoma, breast, cervical and brain/CNS cancers. (iii) To examine the effect of treatment, run on a subset of the exposed group who were diagnosed from 1997 onwards and whose treatment could therefore be established from Scottish Cancer Registry records. The small number of patients for whom the treatment received was recorded as unknown were excluded from this analysis. All models were run in Stata version 14 MP (StataCorp LP, College Station, TX, USA). Model assumptions were checked by splitting follow-up time into three periods and comparing HRs during these periods.

## Results

### All pregnancies following cancer diagnosis

**Figure 1:**
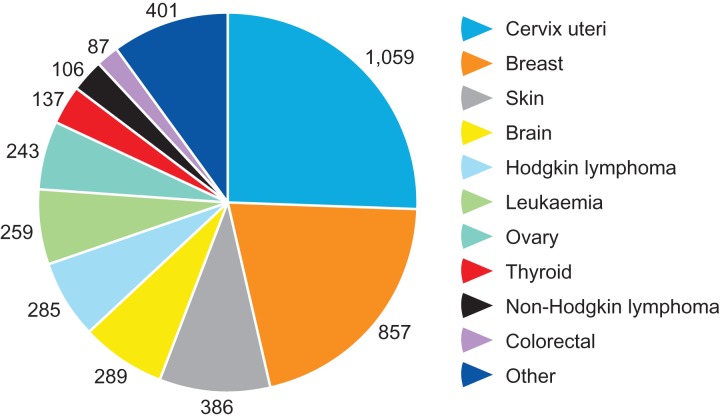
The impact of specific cancer diagnoses on the overall pregnancy deficit. The data shown are the differences between the number of pregnancies expected and those observed for each diagnostic group over the period of this study, represented graphically as a proportion of the total pregnancy deficit. Thus women with cervical cancer aged up to 39 had 1059 fewer pregnancies than matched controls over the period 1984–2014, which was 26% of the total pregnancy deficit.

This analysis included 23 201 women aged 39 or younger at time of cancer diagnosis (Supplementary Information Table S4). Overall the cancer survivors achieved a lower than expected number of pregnancies compared to the general population of women: 6627 observed compared to 10 736 expected pregnancies, SIR 0.62 (95% CI: 0.60, 0.63: Table I). Thus cancer survivors were approximately 38% less likely to achieve pregnancy after diagnosis. SIR was significantly reduced for women with all cancer types with the exception of liver cancer, which was the least prevalent. SIR ranged from 0.34 (0.31–0.37) for women with cervical cancer, to 0.87 (0.84–0.90) for skin cancers (Table I). The contribution of diagnostic groups to the overall pregnancy deficit (Fig. 1) shows that cervical and breast cancer accounted for 26 and 21% of the pregnancy deficit, respectively, with additional substantial contributions (6–9% of the total deficit) from skin cancer, brain/CNS cancers, Hodgkin lymphoma and leukaemia.

**Table I:** Standardized incidence ratio for subsequent pregnancy in all women with cancer onset at age ≤39 years, 1981–2012.

	Women with cancer		Pregnancies following cancer				
	Number	%	Observed	Expected	SIR*	95% CI	
						Lower	Upper
Total	23201	100.0	6627	10736	0.62	0.60	0.63
Type of cancer							
Colorectal	589	2.5	98	185	0.53	0.43	0.64
Liver	63	0.3	11	11	0.96	0.48	1.71
Bone	236	1.0	99	156	0.63	0.52	0.77
Skin (melanoma/non-melanoma)	5252	22.6	2563	2949	0.87	0.84	0.90
Connective and soft tissue	333	1.4	126	177	0.71	0.59	0.85
Breast	5173	22.3	547	1404	0.39	0.36	0.42
Cervix uteri	3498	15.1	552	1611	0.34	0.31	0.37
Ovary	1129	4.9	415	658	0.63	0.57	0.69
Kidney	237	1.0	56	90	0.62	0.47	0.81
Eye	122	0.5	31	66	0.47	0.32	0.67
Brain, CNS	1045	4.5	208	497	0.42	0.36	0.48
Thyroid	926	4.0	499	636	0.79	0.72	0.86
Hodgkin lymphoma	962	4.1	585	870	0.67	0.62	0.73
Non-Hodgkin lymphoma	673	2.9	217	323	0.67	0.58	0.77
Leukaemia	1077	4.6	235	494	0.48	0.42	0.54
Other	1886	8.1	385	608	0.63	0.57	0.70
Age at cancer diagnosis (years)							
0–14	1638	7.1	561	778	0.72	0.66	0.78
15–24	2674	11.5	2052	2984	0.69	0.66	0.72
25–29	3378	14.6	1906	2821	0.68	0.65	0.71
30–34	5926	25.5	1493	2693	0.55	0.53	0.58
35–39	9585	41.3	615	1459	0.42	0.39	0.46
Deprivation category at cancer diagnosis							
1—Least deprived	4671	20.1	1338	2161	0.62	0.59	0.65
2	4451	19.2	1278	1875	0.68	0.64	0.72
3	4690	20.2	1314	2098	0.63	0.59	0.66
4	4760	20.5	1429	2231	0.64	0.61	0.67
5—Most deprived	4629	20.0	1268	2371	0.53	0.51	0.56
Period of cancer onset							
1981–1988	4628	19.9	1294	2422	0.53	0.51	0.56
1989–1996	5765	24.8	1780	3280	0.54	0.52	0.57
1997–2004	6323	27.3	2184	3303	0.66	0.63	0.69
2005–2012	6485	28.0	1369	1732	0.79	0.75	0.83
Record of chemotherapy							
Yes	6274	27.0	1010	2110	0.48	0.45	0.51
No	7107	30.6	2642	3235	0.82	0.79	0.85
Not known	9820	42.3	2975	5391	0.55	0.53	0.57
Record of radiotherapy							
Yes	4557	19.6	655	1525	0.43	0.40	0.46
No	8538	36.8	2862	3587	0.80	0.77	0.83
Not known	10106	43.6	3110	5624	0.55	0.53	0.57

*Standardized for age, deprivation and calendar year of cancer diagnosis; follow up from date of cancer incidence to death or 31 December 2014.

SIR was significantly reduced across all age at diagnosis and deprivation groups, and there were clear effects of both chemotherapy and radiotherapy (Table I). SIR varied strongly by period of cancer diagnosis, being lower for women with cancer diagnosed in the earlier periods: 0.53 (0.51, 0.56) for women diagnosed 1981–1988 compared to 0.79 (0.75, 0.83) for women diagnosed in 2005–2012. Skin cancer became much more frequent over the period of analysis (Table I): on reanalysis after excluding skin cancers, overall SIR was further reduced to 0.52 (0.51, 0.54), however, the effect of period was still very apparent.

### First ever pregnancies following cancer diagnosis

**Figure 2:**
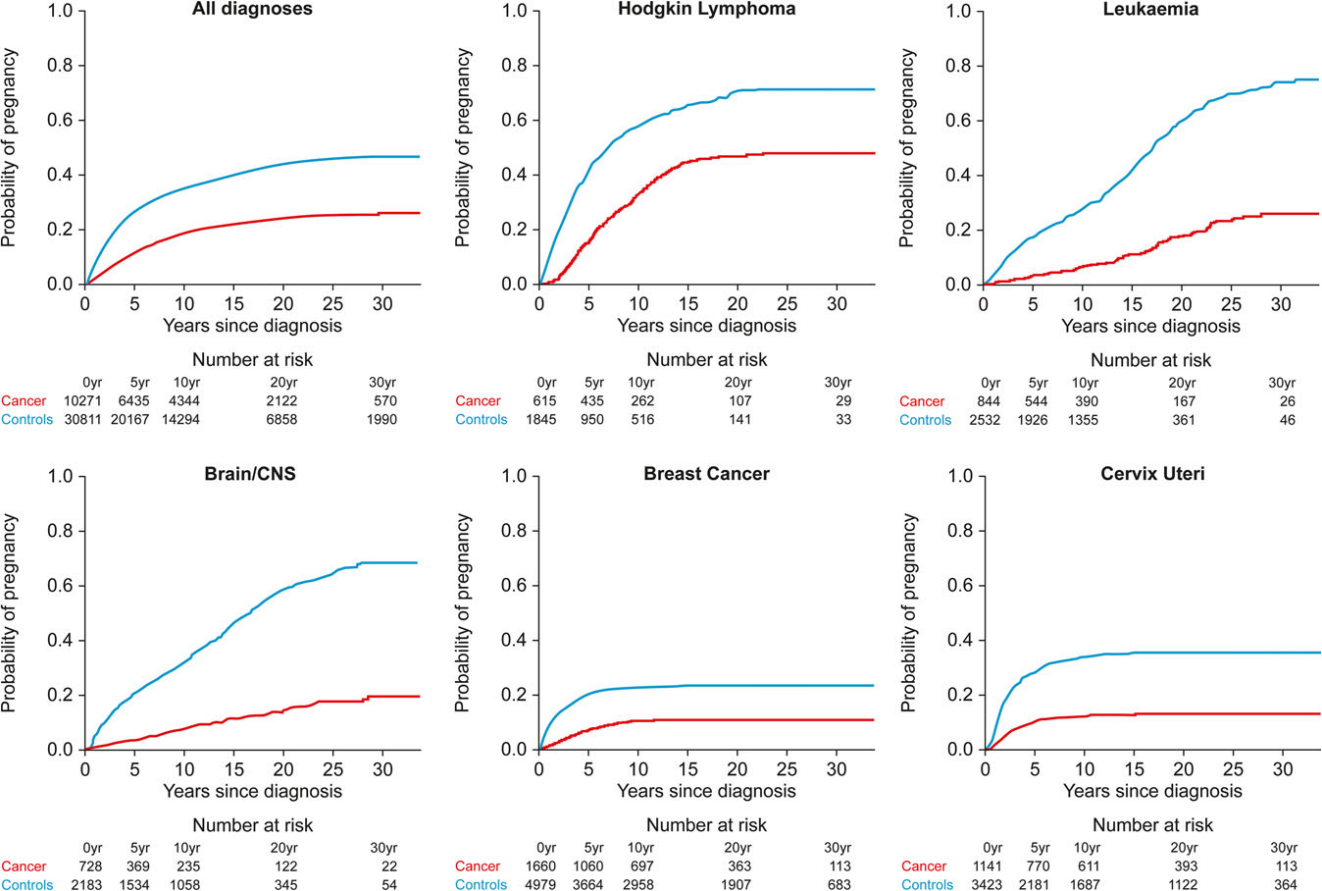
Cumulative probability of first pregnancy after cancer diagnosis (red) in all women with cancer compared to population controls (blue), and in women with breast, cervical, brain/CNS cancers, Hodgkin lymphoma and leukaemia. Tables under each panel indicate the number of women with cancer and controls at the time of diagnosis, and at subsequent time points up to 30 years.

**Figure 3:**
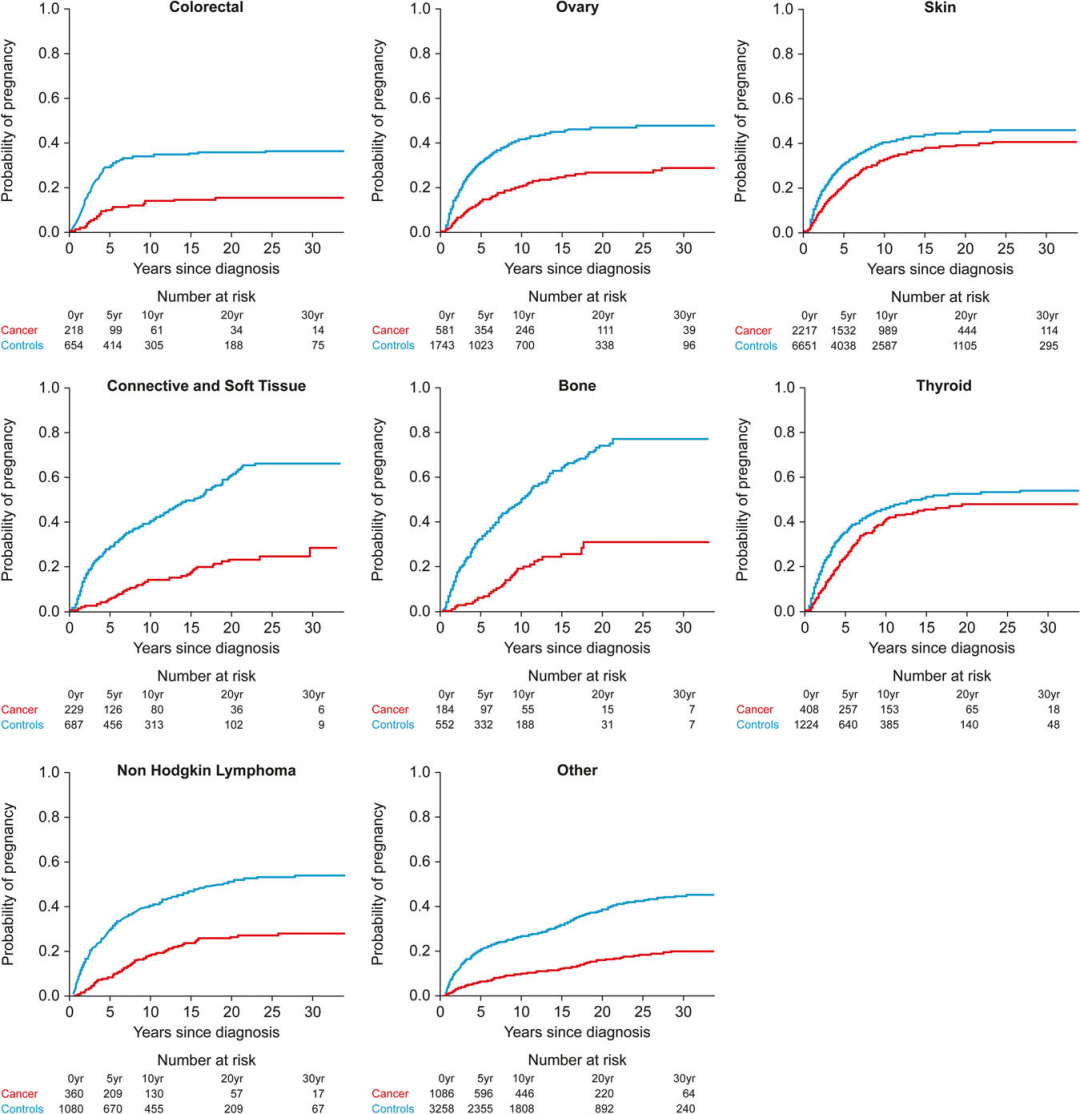
Cumulative probability of first pregnancy after cancer diagnosis (red) in women with diagnoses other than those shown in Fig. 2 compared to population controls (blue). Tables under each panel indicate the number of women with cancer and controls at the time of diagnosis, and at subsequent time points up to 30 years.

This analysis included 10 271 women aged 39 or younger who were nulliparous at the time of their cancer diagnosis and 30 811 matched controls (Supplementary Information Table S5). The cumulative incidence of first pregnancy after cancer was markedly reduced, with overall adjusted HR = 0.57 (95% CI: 0.53, 0.61) for those with >5 years follow-up (Table II and Fig. 2). Adjusted HR was reduced for all diagnostic groups, showing similar general patterns to the SIR analysis with marked impacts of cancers of the cervix (0.22), brain/CNS (0.18) and leukaemia (0.21). Cumulative incidence of subsequent first pregnancy for these diagnoses and for breast cancer and Hodgkin lymphoma (Fig. 2) highlight the very different patterns of subsequent pregnancy compared to age matched controls after these diagnoses (results for other diagnoses are shown in Fig. 3). The impact of previous cancer on subsequent pregnancy was significant for women diagnosed in each age group, with the oldest age group showing a slightly reduced impact (Table II). There were no differences by deprivation category.

**Table II:** Hazard ratio for subsequent first pregnancy in nulliparous women with cancer onset at age ≤39 years, 1981–2012.

	Hazard ratio					
	Unadjusted	95% CI		Adjusted	95% CI	
		Lower	Upper		Lower	Upper
Duration of follow up following cancer diagnosis (1)						
<1 year	0.13	0.11	0.15	0.12	0.10	0.14
1–4 years	0.41	0.39	0.44	0.36	0.34	0.39
≥5 years	0.79	0.74	0.85	0.57	0.53	0.61
Cancer type (2)						
Colorectal	0.28	0.19	0.40	0.26	0.18	0.38
Liver	0.36	0.17	0.78	0.27	0.12	0.60
Bone	0.49	0.37	0.65	0.30	0.22	0.39
Skin (melanoma and non-melanoma)	0.84	0.78	0.90	0.66	0.62	0.72
Connective and soft tissue	0.39	0.30	0.52	0.25	0.19	0.34
Breast	0.20	0.17	0.23	0.30	0.26	0.35
Cervix uteri	0.25	0.21	0.30	0.22	0.18	0.26
Ovary	0.53	0.44	0.62	0.37	0.31	0.45
Kidney	0.34	0.24	0.50	0.33	0.23	0.47
Eye	0.24	0.14	0.42	0.21	0.12	0.37
Brain, CNS	0.24	0.20	0.30	0.18	0.15	0.22
Thyroid	1.03	0.89	1.20	0.69	0.59	0.81
Hodgkin lymphoma	0.91	0.81	1.02	0.46	0.40	0.52
Non-Hodgkin lymphoma	0.47	0.38	0.59	0.34	0.28	0.43
Leukaemia	0.28	0.23	0.33	0.21	0.17	0.25
Other	0.28	0.23	0.34	0.27	0.22	0.32
Age at cancer diagnosis (years) (3)						
0–14	0.39	0.36	0.42	0.37	0.34	0.39
15–24	0.37	0.33	0.41	0.37	0.33	0.41
25–29	0.38	0.35	0.42	0.36	0.33	0.40
30–34	0.39	0.35	0.44	0.39	0.35	0.44
35–39	0.52	0.44	0.62	0.53	0.44	0.63
Deprivation category at cancer diagnosis (4)						
1—Least deprived	0.45	0.40	0.50	0.37	0.34	0.41
2	0.47	0.43	0.53	0.40	0.36	0.44
3	0.44	0.39	0.49	0.36	0.33	0.40
4	0.47	0.42	0.51	0.38	0.35	0.42
5—Most deprived	0.45	0.41	0.50	0.37	0.33	0.41
Period of cancer diagnosis (5)						
1981–1988	0.30	0.27	0.33	0.20	0.18	0.22
1989–1996	0.40	0.37	0.44	0.34	0.31	0.37
1997–2004	0.63	0.58	0.69	0.61	0.56	0.67
2005–2012	0.63	0.57	0.70	0.62	0.56	0.69
Chemo/radiotherapy status (6)						
Chemotherapy only	0.38	0.32	0.45	0.43	0.34	0.53
Radiotherapy only	0.57	0.45	0.74	0.66	0.50	0.86
Chemo and radiotherapy	0.30	0.24	0.37	0.36	0.29	0.47

Models based on 10 271 women with cancer and 30 811 general population controls except for chemo/radiotherapy effects: these models based on 2619 women with cancer receiving chemo/radiotherapy and 2473 not receiving chemo/radiotherapy. Follow up from date of cancer/matching to first pregnancy, death or 31 December 2014.

(1) Hazard ratios relative to controls with same duration of follow-up.

(2) Hazard ratios relative to matched controls for each cancer type.

(3) Hazard ratios relative to controls of same age.

(4) Hazard ratios relative to controls of same deprivation category.

(5) Hazard ratios relative to controls matched in that period.

(6) Hazard ratios relative to patients with cancer who did not undergo chemotherapy or radiotherapy.

**Figure 4:**
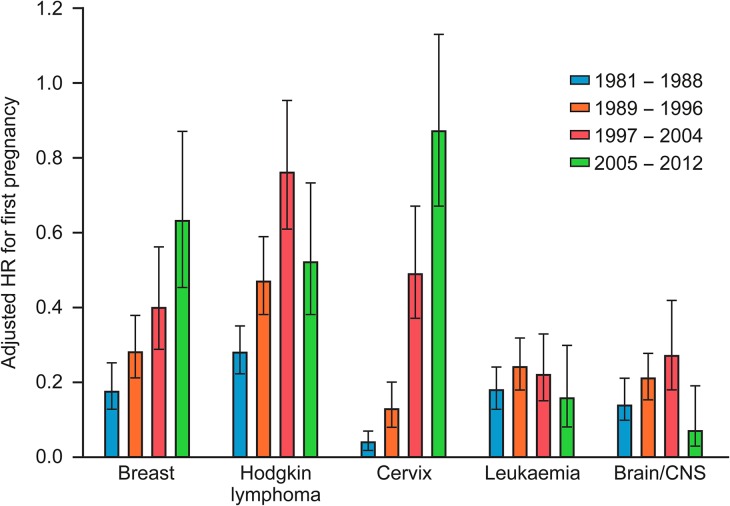
Adjusted HR (with 95% CI) for first pregnancy after cancer diagnosis by period of diagnosis for women with breast, cervical, Hodgkin lymphoma, leukaemia and brain/CNS cancers.

As in the SIR analysis, the impact of cancer was much less for women diagnosed in more recent periods, despite possible limitations of shorter follow up with more recent diagnosis. Among women diagnosed with cancer in 1981–1988, adjusted HR for subsequent first pregnancy was 0.20 (0.18, 0.22) compared with 0.62 (0.56, 0.69) in women diagnosed in 2005–2012. Analysis of the effect of period of diagnosis revealed marked increases in adjusted HR for subsequent first pregnancy in more recent periods for breast cancer, Hodgkin lymphoma and cervical cancer (Fig. 4), with in the latter case women diagnosed in the most recent period showing no significant effect on subsequent first pregnancy (HR = 0.88 (0.68, 1.14)). There was a very different pattern for leukaemia and brain/CNS cancers (Fig. 4), with adjusted HRs remaining very low across all diagnostic periods.

The proportion of first singleton pregnancies after cancer that ended in a termination was lower among women with previous cancer (with analysis by age at diagnosis showing this was significant in all age groups except the oldest) and the proportion ending in a live birth was correspondingly higher (Table III). The proportion of pregnancies achieved that ended in miscarriage or still birth was similar, as was the infant death rate for live births.

**Table III:** Outcomes of singleton first pregnancies among nulliparous women with cancer onset at age ≤39 years, Scotland, 1981–2012 and matched controls.

Singleton first pregnancies following cancer onset/matching date to 31 December 2014	Nulliparous women with cancer		Control women		Difference	95% CI	
	Number	%/rate*	Number	%/rate*		Lower	Upper
Total	2071	100	11772	100			
Miscarriage	203	9.8	1095	9.3	0.5	−0.9	1.9
Termination	231	11.2	1725	14.7	−3.5	−5.0	−2.0
Still birth	8	0.4	53	0.5	−0.1	−0.4	0.2
Live birth	1629	78.7	8899	75.6	3.1	1.1	5.0
Infant death	12	7.4	43	4.8	2.5	−1.9	6.9

*% of all first singleton pregnancies apart from for infant deaths which is per 1000 live births.

## Discussion

This analysis provides robust, population-based evidence for the effect of cancer and its treatment on subsequent pregnancy in women aged under 40 at the time of diagnosis. There was an overall reduction in the likelihood of pregnancy after diagnosis of 38% compared to the general population, with a comparable reduction in the incidence of first pregnancy after cancer. The reduction was seen in all groups by age at diagnosis and across the diagnostic spectrum, even in cancers which are predominantly managed surgically, although treatment with chemotherapy and radiotherapy were both shown to have important effects. There was no marked variation by deprivation index.

The clear reduction in overall impact on subsequent pregnancy by treatment period showed marked differences by diagnosis. The striking change for women with cervical cancer is likely to reflect changes in both the detection and treatment of early stages of cervical cancer, with widespread screening introduced in the 1980s, and the current development of fertility-sparing surgery (Rob *et al.*, 2011). Hodgkin lymphoma and breast cancer also showed improvement in chance of first pregnancy after cancer with more recent diagnosis. This improvement is in keeping with recent data (Bramswig, *et al.*, 2015) showing limited overall impact of Hodgkin lymphoma in girls on the proportion subsequently achieving parenthood (although abdominal radiotherapy was associated with substantial reduction) and shows parallels to overall reductions in secondary mortality in Hodgkin lymphoma and other childhood cancers, associated with reductions in the use of radiotherapy (Armstrong, *et al.*, 2016). The impact of breast cancer and its treatment may be augmented by prolonged adjuvant endocrine therapy in hormone-sensitive disease (Shandley *et al.*, 2017), with the consequent reduction in fertility due to increasing age being of substantial importance, and concern over the potential impact of pregnancy on the risk of recurrence (Azim *et al.*, 2013).

In marked contrast leukaemia and brain/CNS cancers showed no improvement in the chance of subsequent first pregnancy over the 30-year period of analysis. Bone marrow transplantation (BMT) remains the most effective treatment for leukaemia in young people excluding children (Sellar *et al.*, 2011; Rowe, 2013), and our findings reflect the high risk of loss of fertility associated with total body irradiation or high dose alkylating agent based chemotherapy as conditioning treatment before BMT for acute leukaemia (Salooja *et al.*, 2001). For brain tumours effective treatments such as cranio-spinal radiotherapy can impact on later reproductive function (Bath *et al.*, 2001). Survivors of brain/CNS cancer may also have a significant neurocognitive impairment (Ellenberg *et al.*, 2009) and are less likely to be married or cohabiting (Frobisher *et al.*, 2007; Koch *et al.*, 2011), illustrating some of the range of factors that impact on the likelihood of post-cancer pregnancy. Choosing not to have a (further) pregnancy after cancer, i.e. not to complete a previously intended family size, may also have an impact. This may underlie the effect of cancers which are largely managed surgically, such as skin cancers, and would not be expected to have an adverse effect on the reproductive system.

Reassuringly, our results show no increased risk of miscarriage or still birth among first pregnancies achieved after a cancer diagnosis. The slightly lower proportion of pregnancies among women with previous cancer that end in a termination of pregnancy may reflect more active planning of pregnancies in the cancer group, increased use of contraception, or continuation of more unplanned pregnancies: further research is needed to identify which of these factors have a role. Infant death following a live birth was uncommon in both the cancer and control groups, with no evidence of increased risk among the offspring of women with cancer. Although the present data show no reduction in the chance of live birth after cancer, previous studies have indicated that cancer survivors are at risk of a range of pregnancy complications including miscarriage, premature delivery and low birth weight, particularly associated with radiotherapy to a field that includes the pelvis for childhood cancer (Clark, *et al.*, 2007; Winther *et al.*, 2008; Reulen, *et al.*, 2009; Signorello *et al.*, 2010; Haggar, *et al.*, 2014; Reulen *et al.*, 2017). The finding of a reduced prevalence of termination of pregnancy differs from a Danish analysis (Winther *et al.*, 2009b); this may reflect both the size of the present analysis, and the accurate recording of this outcome.

The varied and changing but still reduced chance of pregnancy in young female survivors of cancer demonstrated here means that it remains important to identify those girls and women at significant risk to offer timely access to fertility preservation treatments (Anderson *et al.*, 2015). These include oocyte vitrification in postpubertal women, although this requires ovarian stimulation, with significant time implications (Argyle *et al.*, 2016; Lavery *et al.*, 2016), or cryopreservation of ovarian tissue which has no lower age limit but requires a surgical intervention and may risk re-introducing malignant cells (Loren, *et al.*, 2013). Appropriate longer term follow-up for those young females at risk of a reduced chance of a pregnancy after successful treatment of their primary cancer remains important (Scottish Intercollegiate Guidelines Network, 2013). Reproductive counselling, diagnosis and treatment of POI and access to assisted reproductive technologies should be a priority for young female cancer survivors who are deemed to be at high risk of a reduced chance of pregnancy (Wallace *et al.*, 2012).

The largest datasets on pregnancy after cancer come from studies of childhood cancer survivors, with little comparable data from women diagnosed in adulthood. The CCSS has provided benchmark studies demonstrating this risk (Sklar *et al.*, 2006; Green, *et al.*, 2009) although it is not population based. The relative risk for female survivors of ever being pregnant was 0.81 compared with siblings (Green, *et al.*, 2009), and a more recent analysis reported a hazard ratio for pregnancy of 0.87 in females who had been treated with chemotherapy but not radiotherapy to the brain or pelvis (Chow, *et al.*, 2016). The BCCSS (Reulen, *et al.*, 2009) also reported that the number of live births observed from all female survivors was two-thirds of that expected, though that analysis is limited to girls aged under 15 years at diagnosis. A recent population based Swedish cohort study (Armuand, *et al.*, 2017) of 552 female survivors of cancer in childhood or adolescence (<21 years) who were born between 1973 and 1977 showed that the HR for first live birth was 21% lower than for controls, with particular effects of CNS tumours and leukaemia. Our study, includes all girls and women diagnosed with cancer without potentially biased incomplete or selective follow-up and includes those who have received radiotherapy to a field that includes the brain or pelvis. This suggests a greater impact with adjusted HR of 0.37 for girls and young women in both the under 15 years and 15–24 age group. The present analysis now provides for the first-time robust analysis of the effect of cancer on the likelihood of a pregnancy, and of a first pregnancy, after all cancer diagnoses in girls and adult women, up to the age of 39.

While major strengths of this analysis are its size and unbiased, population based data, and the inclusion of women up to age 39, weaknesses include the necessarily short duration of follow-up of those most recently diagnosed, and the lack of detailed treatment information. The effect of cancer was confirmed here to be most marked in the early years after diagnosis, so the HR for those diagnosed in the most recent period will be a conservative analysis. Treatment information is at present not routinely collected in the national databases used in this study, but is important to allow more precise analysis of the effects of components of treatment regimens on fertility. The relative contributions of diagnosis/treatment related loss of fertility and psychological and social factors such as concerns over cancer recurrence and other health issues (Schover, 2005; Howard-Anderson, *et al.*, 2012; Nilsson *et al.*, 2014) are unclear. Information on early miscarriage is also likely to be incomplete, as the data only include miscarriages managed in a hospital setting, but this is likely to affect data from cancer survivors equally to the general population.

In conclusion, this study shows the impact of cancer on the subsequent chance of pregnancy, both overall and of first pregnancy, in girls and women. A reduction in the chance of first pregnancy was seen across all ages at diagnosis and widely across diagnostic groups. We clearly show that the impact of cancer on the chance of subsequent pregnancy in young women is much <20–30 years ago for some key diagnoses but remains present, and there has been no improvement in the impact on later pregnancy of other diagnoses, notably leukaemia and brain/CNS cancer. These data quantify the impact of cancer on subsequent pregnancy. They highlight the need for interventions to protect fertility in girls and young women with cancer at the time of diagnosis and to support women considering pregnancy once treatment is completed.

## Authors’ roles

R.A.A.: study design, interpretation, drafting and finalizing article; D.H.B.: study design, data analysis and interpretation, drafting and finalizing article; S.N.: data analysis, study design, interpretation, drafting and approval final article; R.W.: study design, data analysis and interpretation, drafting and finalizing article; C.F.: data analysis and interpretation, drafting and finalizing article; T.W.K.: data analysis and interpretation, drafting and finalizing article; W.H.B.W.: study design, interpretation, drafting and finalizing article.

## Funding

NHS Lothian Cancer and Leukaemia Endowments Fund. Part of this work was undertaken in the MRC Centre for Reproductive Health which is funded by the MRC Centre (Grant MR/N022556/1). The funders had no role in the design, analysis or interpretation of the data.

## Conflict of interest

R.A.A. has participated in Advisory Boards and/or received speakers fees from Beckman Coulter, IBSA, Merck and Roche Diagnostics. He has received research support from Roche Diagnostics, Ansh labs and Ferring. The other authors have no conflicts to declare.

## Supplementary Material

Supplementary Table 1

Supplementary Table 2

Supplementary Table 3

Supplementary Table 4

Supplementary Table 5
