# Supplementary material for: The impact of cancer on subsequent chance of pregnancy: a population-based analysis
Source: Hum Reprod. 2018 Jun 15;33(7):1281–90. doi: 10.1093/humrep/dey216 (PMC6012597; doi:10.1093/humrep/dey216)
Supplement: Supplementary Table 1 [file dey216suppl_table1.pdf]

Human Reproduction, 2018
doi:10.1093/humrep/dey216

human reproduction

SUPPLEMENTARY DATA

Supplementary Table SI International Statistical Classification of Diseases and Related Health Problems 10th Revision (ICD-10) diagnostic codes used to identify included cancers within the Scottish Cancer Registry.

Cancer	ICD-10 Codes*
Colorectal	C18–C20
Liver	C22
Bone	C40–C41
Skin (melanoma and non-melanoma)	C43, C44
Connective and soft tissue	C47, C49
Breast	C50
Cervix uteri	C53
Ovary	C56
Kidney	C64
Eye	C69
Brain, CNS	C70–C72, C75.1–C75.3
Thyroid	C73
Hodgkin lymphoma	C81
Non-Hodgkin lymphoma	C82–C85, C96
Leukaemia	C90.1, C91–C95
Other	All other codes in the range C00–C96
All combined	C00–C96

*Records entered before the introduction of ICD-10 have been forward mapped, using the combination of ICD-9 code and morphology codes.
