# Supplementary material for: The impact of cancer on subsequent chance of pregnancy: a population-based analysis
Source: Hum Reprod. 2018 Jun 15;33(7):1281–90. doi: 10.1093/humrep/dey216 (PMC6012597; doi:10.1093/humrep/dey216)
Supplement: Supplementary Table 2 [file dey216suppl_table2.pdf]

Supplementary Table SII Data sources and ICD codes used to identify pregnancies and their outcomes. Miscarriages, terminations, live births and stillbirths were identified in the national maternity hospital day case and inpatient discharge database (Scottish Morbidity Record (SMR) 02) using the 'condition on discharge' variable. In addition, miscarriages and terminations managed in early pregnancy/gynaecology services were identified in the national general hospital day case and inpatient discharge database (SMR01) using the following ICD-9 and 10 diagnostic codes.

Pregnancy outcome	ICD-9	ICD-10
Miscarriage	630–634, 637	O00–O03, O05, O06
Termination	635–636, 638	O04, O07
