# Supplementary material for: The impact of cancer on subsequent chance of pregnancy: a population-based analysis
Source: Hum Reprod. 2018 Jun 15;33(7):1281–90. doi: 10.1093/humrep/dey216 (PMC6012597; doi:10.1093/humrep/dey216)
Supplement: Supplementary Table 3 [file dey216suppl_table3.pdf]

Supplementary Table SIII **ICD diagnostic and Office of Population Censuses and Surveys Classification of Interventions and Procedures (OPCS) procedural codes used to identify cancer treatment status within the national general hospital day case and inpatient discharge database (SMR01) for patients diagnosed with cancer prior to 1997.**

Treatment	ICD-9	ICD-10	OPCS-3	OPCS-4
Chemotherapy	V581	Z511,Z512	083, 408, 8882, 9611–9613	T133, T482, X353, X353, X72, X73
Radiotherapy	V580	Z510	990–999	A107, A613, B022, C242, C395, C455, C823, C824, J123, J487, M706, M712, P064, P205, Q151, T481, Y363, Y364, Y902, X65, Y35, Y91

Date ranges
- ICD-9:1981 to March 1996 (December 1996 in cancer registration data).
- ICD-10: April 1996 (January 1997 in cancer registration data) to present.
- OPCS-3: 1977–1988.
- OPCS-4: 1989 to present.
