# Supplementary material for: The impact of cancer on subsequent chance of pregnancy: a population-based analysis
Source: Hum Reprod. 2018 Jun 15;33(7):1281–90. doi: 10.1093/humrep/dey216 (PMC6012597; doi:10.1093/humrep/dey216)
Supplement: Supplementary Table 4 [file dey216suppl_table4.pdf]

Supplementary Table SIV **Number of women with cancer onset at age ≤39 years, 1981–2012, Scotland: cancer type by period of diagnosis.**

Type of first cancer	Period of cancer onset			
	1981–1988	1989–1996	1997–2004	2005–2012
Colorectal	122	150	145	172
Liver	10	17	15	21
Bone	54	54	69	59
Skin (melanoma and non-melanoma)	743	1195	1575	1739
Connective and soft tissue	68	98	86	81
Breast	1094	1316	1443	1320
Cervix uteri	850	894	844	910
Ovary	224	261	328	316
Kidney	35	67	57	78
Eye	38	31	30	23
Brain, CNS	227	314	232	272
Thyroid	138	206	258	324
Hodgkin lymphoma	245	225	260	232
Non-Hodgkin lymphoma	139	177	180	177
Leukaemia	233	269	310	265
Other	408	491	491	496
Total	4628	5765	6323	6485
