# Supplementary material for: The impact of cancer on subsequent chance of pregnancy: a population-based analysis
Source: Hum Reprod. 2018 Jun 15;33(7):1281–90. doi: 10.1093/humrep/dey216 (PMC6012597; doi:10.1093/humrep/dey216)
Supplement: Supplementary Table 5 [file dey216suppl_table5.pdf]

Supplementary Table SV **Nulliparous women diagnosed with cancer aged ≤39 years in Scotland 1981–2012 and general population controls.**

	Number	%	Person years of follow up	Number of subsequent first pregnancies	Number of subsequent deaths (prior to first pregnancy)
Women with cancer	10271	100	113 080	2114	2721
General population controls	30811	100	362 899	11915	570
Women with cancer only					
Age at cancer diagnosis (years)					
0–14	1635	15.9	18 317	272	463
15–24	2042	19.9	18 825	755	396
25–29	1599	15.6	14 064	564	309
30–34	1963	19.1	22 570	374	486
35–39	3032	29.5	39 304	149	1067
Deprivation category at cancer diagnosis					
1—Least deprived	2182	21.2	25 110	432	522
2	2002	19.5	21 628	417	519
3	2051	20.0	22 705	409	578
4	2133	20.8	23 326	457	555
5—Most deprived	1903	18.5	20 311	399	547
Period of cancer diagnosis					
1981–1988	2601	25.3	44 802	446	1043
1989–1996	2431	23.7	31 344	594	776
1997–2004	2487	24.2	23 632	632	526
2005–2012	2752	26.8	13 301	442	376
Cancer type					
Colorectal	218	2.1	1823	28	104
Liver	38	0.4	185	6	20
Bone	184	1.8	1448	41	76
Skin (melanoma/non-melanoma)	2217	21.6	25 289	750	116
Connective and soft tissue	229	2.2	2075	43	98
Breast	1660	16.2	19 029	161	673
Cervix uteri	1141	11.1	15 976	139	269
Ovary	581	5.7	6377	134	139
Kidney	142	1.4	1695	24	37
Eye	89	0.9	1459	11	14
Brain, CNS	728	7.1	6314	85	348
Thyroid	408	4.0	4243	159	16
Hodgkin lymphoma	615	6.0	6720	235	73
Non-Hodgkin lymphoma	360	3.5	3397	77	124
Leukaemia	844	8.2	9184	112	261
Other	817	8.0	7865	109	353
Record of chemotherapy					
Yes	2733	26.6	20 978	343	902
No	2850	27.7	20 635	780	243
Not known	4688	45.6	71 468	991	1576
Record of radiotherapy					
Yes	1679	16.3	14 207	210	613
No	3754	36.5	26 845	864	471
Not known	4838	47.1	72 027	1040	1637
